# Insanity and Its Relations to Crime

**Published:** 1873-10

**Authors:** 


### Books Reviewed.

*Insanity and its Relations to Crime.* A Text and a Commentary. By William A. Hammond, M. D., etc. New York: D. Appleton & Co., 1873.

A portion of this essay was published in *Putnam's Magazine* for September, 1870, under the title " Society *versus* Insanity, and at that time attracted considerable attention. The greater portion is now published, however, for the first time.

The essay is divided into two chapters. Chapter first, the text, gives the details of three homocides in which the plea of Insanity was advanced at the trial, These cases are taken from French sources.

Case I. is an account of an unprovoked murder of a girl of the Canton Ferte Aleps by Antoine Leger. The details of this crime are particularly atrocious, and these together with the statements and actions of the prisoner after his arrest, point to a lack of mental equilibrium.

We give two of the questions propounded to the prisoner, and his answers, showing that he was conscious of having committed a crime:

Q. You were then agitated by fear. You felt that you had done wrong.

A. Yes; when I recovered consciousness, I went and hid myself in the rocks, and could not sleep; I had not my mind about me; the next day I walked to the fields beyond the hills; I washed myself with water which I found in the rocks, and I also washed my shirt: I cut off the collar and the sleeves, which were bloody; there I met one of the guard, and I fled; when I saw any person I hid myself; the guard cried, "Halt, in the name of the king!" and I was at once arrested.

Q. What do you wish to say about the young Debully?

A. I was unconscious; I was urged by the evil spirit.

In reply to a question asking if he had often conceived the idea of carrying a woman to the cave, he says: "I had the idea, but I did not do it. Despair led me to take up my abode there; my mind was gone." He also describes the manner in which he attacked the girl and carried her to his cave. The jury failed to find the man insane, and rendered a verdict of guilty. He received the sentence of death, and was accordingly executed. The *post mortem* examination revealed undubitable evidence of disease of the brain.

The second case is that of the man Jobard, who at the theatre at Lyons stabbed a young lady, who sat immediately in front of him, to the heart. When arrested he appeared perfectly calm, and declared that he did not even know his victim; that he had killed her to be killed in return. Having led a life of depravity though seemingly exemplary in his conduct, he became disgusted with himself, and resolved to get rid of life. He could not think of suicide, for that would cause him to appear before God loaded with sins. He therefore resolved to do something which would cause him to be condemned to death by the law. He would thus have sufficient time for repentance, and was confident of the pardon of God. He had endeavored to do as little harm as possible, and had therefore refrained from killing a depraved person, who would thus be sent unprepared for death into the presence of God, and had accordingly selected an apparently virtuous victim.

This man states that he has contracted many grave vices, from which he was powerless to abstain. He assumes that it is impossible to reform, and yet is conscious that he must discontinue his course of depravity. On a second interrogation he declares that he had always understood that he was accountable both to God and man for his crime. He acknowledges the liberty of acting freely; says that he would have stopped had he comprehended the falsity of his reasoning; that could he have been advised by some one he never would have committed the deed.

In regard to the sanity of this man there was a diversity of opinion among the experts called to examine him. The conclusions of Dr. Gensoul and MM. Gromier and Travernier were: "1. That at the moment of committing the murder Jobard was suffering from a paroxysm of homocidal mania. 2. That he ought not to be considered responsible for an act done without the participation of his normal will. 3. But as this kind of insanity is dangerous to society, society has the right to put Jobard in such a position as will render it impossible for him to do further harm, and that therefore he should be placed for life in a lunatic asylum."

Jobard was, however, indicted and tried for murder with premeditation. After a long trial the jury rendered a verdict of guilty as to the homocide and premeditation, but with extenuating circumstances. He was condemned to hard labor for life. His sentence was afterward mitigated to a considerable extent. He remained incapable of fixing his attention, but had no further exacerbation of his malady.

The third and last case is that of Jules ——, a young man of nineteen, who had for several weeks entertained ideas of suicide. After dining with his father and step-mother, he goes out to take a stroll, when he is seized by his suicidal mania, which is as suddenly changed into a desire to kill his step-mother. He returns to his brother's room, takes two pistols, and descending to the dining-room, discharges one of them at his step-mother, with instant fatal effect. His father at once attempts to seize him, when he rushes from the house crying: "I am a madman, an idiot; I have killed my step-mother." He afterward surrenders himself to the police, to whom he related all the circumstances of his crime. He was acquitted on the plea of insanity. In his account of the act he affirms that he was actuated by an irresistable force, which dragged him in spite of himself. He says: "If my father had addressed to me one word when I entered the drawing-room, a single word, whatever it might have been, I should not have killed my step-mother." Five years subsequent to the murder he committed suicide over the grave of his victim.

We have dwelt to some length upon these cases cited by the author for the reason that they are as tpyical as any of the relations of insanity to crime, and that they form the basis of what follows in the commentary. The writer says, in commenting on these cases: "Certainly Leger was as insane as Jobard or Jules, and yet he was executed. Certainly Jules was as responsible as either Leger or Jobard, and he was acquitted. Certainly Jobard was no more insane than Leger, nor more responsible than Jules, and yet he was sent to the galleys for life. Such inconsistencies show the great need of a fixed and definite principle by which all juries should be governed, and this is, I think, no difficult matter to establish." We shall endeavor to give further on the author's views in regard to this principle.

Sin and crime are not to be viewed as the same thing; morally, between man and God, the intention constitutes the sin; the crime is measured by the injury done to society. Justice and law in this view are seen to be different. A law may be unjust as regards an individual, or a few individuals, and beneficial to society at large; it is then a good law. It may be just to the individual and injurious to society; it is then a bad law. What society requires is protection, and it has no business with abstract justice Justice is to be inforced between man and man by law, not between society and man, unless it is consistent with the great principle of protection—the principle of the greatest good to the greatest number.

The object of punishment, then, is the protection of society, the reformation of the criminal being usually lost sight of. This safety is secured in two ways. 1. By the effect which it has upon the individual in intimidating him, or by placing him in such a condition that it will be impossible for him for a limited period, or ever again to break the law. 2. By the example which it affords to others who might feel inclined to commit crimes, but who are kep in check by the certainty or probability of punishment. Our author cites the facts that a criminal is punished even when he is totally ignorant of the existence of the law which he has broken, and that in the most enlightened country—except our own—the heirs of a traitor are made to forfeit the property which would naturally fall to them, and are thus punished for a crime wholly beyond their control and perhaps knowledge; that the Bible states that the sins of the fathers shall be visited upon the children. He considers that from a similar point of view no valid argument can be adduced against the punishmunt of the insane, even though they are morally irresponsible for their acts by reason of delirium, dementia, morbid impulse, emotional insanity, or any other form of mental aberration. The writer seems to us in danger, in his anxiety to prove his point, of starting from a wrong basis. He lays down as the object of punishment the safety of society, which is to be secured in two ways, as above. What effect punishment may have upon an insane criminal, who is incapable of appreciating the crime which he has committed, and hence the object of his punishment, we are unable to say; certainly we think not much. As for placing him in a condition in which he will be incapable of doing further harm, it is certainly admitted that all insane persons, whether possessed of an irresistible homocidal impulse or not, are better both for their own good and that of society, placed in a state of sequestration. He argues that the force of example is strong in those of an unbalanced or weak mind, and that the punishment of those who are led through mental aberration to commit crime, will exercise a restraining influence upon others who might be tempted to imitate them. It has certainly been noticed, and we have had numerous and recent examples in our own city, that if a man commits suicide in some unusual or startling manner, he is straightway followed by others, who seem to be led on by the force of example, and whose minds after brooding over the details of the affair are too weak to resist the impulse toward self-destruction. How powerful the influence for good the punishment of an insane criminal might be made to be upon those who might otherwise be led to imitate him is unknown; it certainly seems that it might have some effect. The question is, are we ready for this reform in our code of laws? Is the public capable of appreciating the beneficial effects which might arise from such a course of action?

Dr. Hammond says: "The punishment awarded to the insane should be apportioned to the nature of the crime and the character of the insanity, and should thus extend from simple sequestration to fine and imprisonment, with labor, and in some cases even death, so long as death is by law the punishment for certain kinds of homocide. The only form of insanity which, in my opinion, should absolve from responsibility, and, therefore, from any other punishment except sequestration, are such a degree of idiocy, dementia, or mania, as prevents the individual from understanding the consequences of his act, and the existence of a delusion in regard to an act or matter of fact which, if true, would justify his act."

Dr. Hammond's views are what might be called conservative; they do not represent the ideas of the majority of experts on insanity. Some of them will, in time, be accepted, others will prove to be fallacious. Much can be learned from studying his essay, and it will doubtless awaken a new train of thoughts in the minds of his readers. In conclusion, we copy the following from page 75:

"Insanity is only a manifestation of disease of the brain. Its basis is as much physical as is that of pneumonia, or valuvlar disease of the heart, or any other affection which all regard as bodily. It is no more possible for a person to be insane without other evidences of disease than mental derangement, than for pneumonia to exist with no other symptoms than disturbed respiration, or for valvular disease of the heart to be restricted in its manifestations to irregularity of the circulation of the blood. The doctrine that an individual can be entirely sane immediately before and after any particular act, and yet insane at the instant the act was committed, is contrary to every principle of sound psychological science. Even in the most striking instances of what is called transitory mania, or morbid impulse, the evidences of pre-existent and subsequent disease of the brain, will be found if they are looked for with skill and diligence and intelligence."

